# Generation and modulation of catalytically relevant states of a dye-decolourizing peroxidase using time-resolved serial femtosecond crystallography with drop-on-chip mixing and X-ray-driven reduction

**DOI:** 10.1107/S2059798326001658

**Published:** 2026-04-07

**Authors:** Marina Lučić, Johan Glerup, Pierre Aller, Danny Axford, Nicholas Devenish, Jaehyun Park, Anastasiia Shilova, Arturo Landeros de la Isla, Richard W. Strange, Tiankun Zhou, Robin L. Owen, Jonathan A. R. Worrall, Michael A. Hough

**Affiliations:** a School of Life Sciences University of Essex Wivenhoe Park ColchesterCO4 3SQ United Kingdom; b Diamond Light Source Harwell Science and Innovation Campus DidcotOX11 0DE United Kingdom; c Research Complex at Harwell Rutherford Appleton Laboratory DidcotOX11 0FA United Kingdom; d Pohang Accelerator Laboratory, POSTECH, Pohang, Gyeongbuk37673, Republic of Korea; Institut de Biologie Structurale, France

**Keywords:** heme enzymes, time-resolved crystallography, peroxidases, ferryl heme, radiation damage, serial synchrotron crystallography

## Abstract

Metalloenzymes containing a heme cofactor catalyse a wide range of oxidative reactions critical to life. Understanding the structure and electronic states of the heme across the catalytic cycle is essential in understanding the oxidative chemistry performed on the substrate. This work demonstrates *in crystallo* manipulation of the heme-iron oxidation state in a B-type dye-decolourizing peroxidase from *Streptomyces lividans* (DtpB) using multiple, complementary, serial crystallography approaches. Fixed-target drop-on-chip serial femtosecond crystallography (SFX) together with dose-resolved serial synchrotron crystallography (SSX) allowed DtpB to be driven between multiple iron oxidation states. Drop-on-chip addition of hydrogen peroxide with fixed-target SFX is used to generate a ferryl [Fe(IV)=O] species, while the X-ray-driven approach modulates the iron oxidation state, with an apparent two-electron reduction leading to a return to a ferric state. The formation and dose response of the Fe(IV)—O state is highly variable between the chemically identical heme groups of the DtpB hexamer, highlighting the importance of understanding the effect of the crystalline lattice on observed changes in time- and dose-resolved crystallography.

## Abstract 2

We characterize the formation of a ferryl state of a dye-decolourizing peroxidase by drop-on-chip serial femtosecond crystallography and also the modulation of the heme redox state using dose-resolved serial synchrotron crystallography.

## 1. Introduction

Heme enzymes carry out a wide range of oxidative chemistries using high-valent heme–oxygen species that are formed through the two-electron reduction of molecular oxygen (O_2_) or hydrogen peroxide (H_2_O_2_) (Poulos, 2014 ▸). A significant challenge in structurally characterizing the reaction mechanisms of heme enzymes has been the typically transient nature of the oxidizing high-valent ferryl [Fe(IV)] intermediates Compound I and Compound II (Moody & Raven, 2018 ▸). Furthermore, these ferryl intermediates, as well as the ferric state, are extremely susceptible to electronic state changes when exposed to an X-ray beam, as X-ray-generated solvated electrons rapidly result in site-specific radiation damage (Kekilli *et al.*, 2017 ▸; Pfanzagl *et al.*, 2020 ▸). These X-ray-induced changes occur extremely quickly: in significantly less time (dose) than that required to obtain a crystallographic dataset (Garman & Weik, 2023 ▸). Obtaining crystallographic structures of intact or near-intact ferryl heme states has required novel approaches such as cryo-trapping followed by composite data collection from multiple crystals together with single-crystal spectroscopies (Berglund *et al.*, 2002 ▸; Gumiero *et al.*, 2011 ▸; Chreifi *et al.*, 2016 ▸; Kwon *et al.*, 2021 ▸; Lučić, Chaplin *et al.*, 2020 ▸), neutron diffraction (Casadei *et al.*, 2014 ▸; Kwon *et al.*, 2016 ▸) or serial approaches such as serial synchrotron crystallography (SSX) or serial femtosecond crystallography (SFX) (Lučić, Svistunenko *et al.*, 2020 ▸; Lučić *et al.*, 2022 ▸; Worrall & Hough, 2022 ▸).

**Figure 1:**
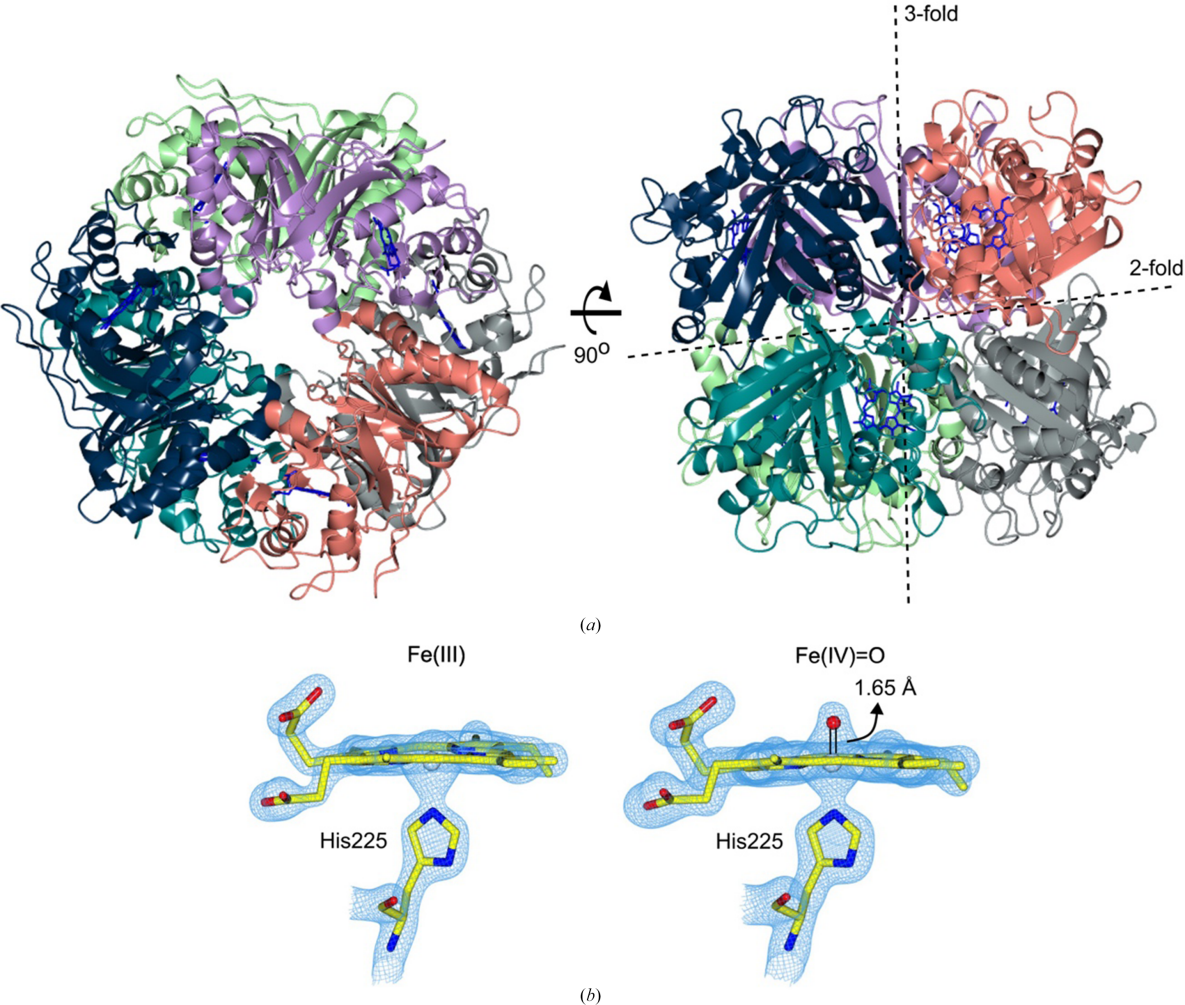
The SFX structure of *S. lividans* DtpB. (*a*) The ferric DtpB hexamer shown in cartoon representation possesses *D*3 dihedral symmetry with the rotation axis indicated. Each of the six chains (*A*–*F*) is colour-coded: *A*, navy blue; *B*, grey; *C*, teal; *D*, mint green; *E*, coral; *F*, lavender. The heme group in each chain is shown in blue sticks. (*b*) The active-site heme of chain *A* in the pentacoordinate ferric, Fe(III), state and the H_2_O_2_-reacted [Fe(IV)=O] Compound I state. 2*F*_o_ − *F*_c_ electron-density maps are contoured at 1.5σ (blue mesh) with heme and the proximal His ligand shown in sticks. The modelled O atom is shown as a red sphere with bond length indicated. [PDB entries 6yrj (ferric) and 6yrd (Compound I).]

The dye-type peroxidase B (DtpB) is a heme peroxidase discovered in *Streptomyces lividans* that belongs to the B-type subfamily of dye-decolorizing peroxidases (DyPs). Its functional role is unknown, but in addition to facilitating the conversion of harmful peroxide compounds to less toxic substances, it can also bind nitric oxide (NO) and therefore could play a role in NO signalling pathways (Smyth *et al.*, 2025 ▸). A unique feature of DtpB is that it has the relatively unusual property of possessing a long-lived Compound I species consisting of a Fe(IV)–oxo porphyrin π-cation radical, which is stable at room temperature over several hours (Lučić, Svistunenko *et al.*, 2020 ▸). Safely storing highly reactive oxidizing equivalents in this way until a reducing substrate becomes available may indeed be a key requirement in its physiological function (Lučić *et al.*, 2024 ▸). On mixing ferric DtpB microcrystal slurries with H_2_O_2_, brown DtpB crystals turn green, indicative of Compound I formation, and remain green for several hours (Lučić, Svistunenko *et al.*, 2020 ▸). Taking advantage of this long-lived Compound I species, we previously used fixed-target SFX to obtain the first room-temperature Compound I X-ray structure (Lučić, Svistunenko *et al.*, 2020 ▸). DtpB is a hexamer assembly (dimer of trimers) in both the solution and crystalline states (Lučić, Svistunenko *et al.*, 2020 ▸; Lučić *et al.*, 2024 ▸), and is assembled from identical subunits comprising of an α+β dimeric fold each containing a *b*-type heme that is co-ordinated on the proximal face by a His residue (Fig. 1 ▸). No cooperativity between the subunits on reacting with H_2_O_2_ is apparent (Lučić, Svistunenko *et al.*, 2020 ▸; Lučić *et al.*, 2022 ▸, 2024 ▸). In the ferric Fe(III) state, the SFX structure revealed an absence of water molecules on the distal side of the heme (Lučić, Svistunenko *et al.*, 2020 ▸), where H_2_O_2_ binds to the heme Fe and undergoes O—O bond heterolysis to form the Fe(IV)–oxo porphyrin π-cation radical (Compound I) with release of a H_2_O molecule (Fig. 1 ▸*b*). Having a ‘dry’ heme has subsequently been shown to influence the electron-transfer reactivity (and stability) of Compound I (Lučić *et al.*, 2022 ▸). In a dry site, the reduction of Compound I is coupled to proton uptake, resulting in a highly unstable Compound II (lowly populated) that rapidly decays to the ferric state. Thus, the chemistry of Compound I in a dry site is ideally suited to the rapid and almost simultaneous delivery of two electrons (Lučić *et al.*, 2022 ▸).

Serial crystallography enables room-temperature time-resolved structural studies of ligand binding and the capture of short-lived reaction intermediates on a reaction pathway (Orville, 2020 ▸; Pearson & Mehrabi, 2020 ▸; Hough & Owen, 2021 ▸). The serial approach provides the advantage of minimizing the effect of radiation damage in synchrotron experiments (SSX) as the dose required for structure determination can be spread over many thousands of crystals, or radiation damage can be essentially ignored through use of SFX at an XFEL source (Williams *et al.*, 2025 ▸). Reactions can be initiated in crystals by *in situ* mixing of microcrystals with substrates or reagents to initiate reactivity (mix-and-diffuse), followed by time-dependent delivery to an X-ray source (Pearson & Mehrabi, 2020 ▸; Caramello & Royant, 2024 ▸). In this way, the possibility exists to obtain atomic-resolution structures of short-lived reaction species along a reaction coordinate to create a stop-motion molecular movie of ligand binding or catalysis (Rabe *et al.*, 2021 ▸). This room-temperature approach may be used in parallel with methods that use cryo-trapping of intermediates to allow data collection by neutron diffraction (allowing visualization of protonation states) or the SF-ROX approach where multiple still diffraction patterns are taken at the XFEL at many positions and orientations of large cryo-trapped crystals (Casadei *et al.*, 2014 ▸; Kwon *et al.*, 2016 ▸, 2021 ▸; Halsted *et al.*, 2018 ▸, 2019 ▸; Hirata *et al.*, 2014 ▸; Suga *et al.*, 2015 ▸).

Several different methods have been developed to mix microcrystals and substrate to initiate reactions prior to serial data collection. These can include mixing jets (Calvey *et al.*, 2019 ▸), mixing within microfluidic chips (Stubbs *et al.*, 2024 ▸) or deposition of a mixed slurry onto a moving tape (Beyerlein *et al.*, 2017 ▸). Perhaps the most well developed approaches are those which use droplets on demand as part of a tape-drive system. In the tape-drive approach droplets containing microcrystals are transferred acoustically onto a moving tape, and typically much smaller droplets containing a substrate are then fired into the crystal-containing droplets to initiate the reaction at a particular time interval before the droplet on the tape intersects with the beam. This approach has been used with great success in time-resolved SFX experiments at the LCLS (Butryn *et al.*, 2021 ▸).

An attractive alternative implemented both at synchrotron and XFEL beamlines exploits the well established fixed-target serial delivery approach and combines this with the addition of droplets containing substrates to the individual wells containing microcrystals, again at a defined time point before the measurement of diffraction data. The first implementation of this was named LEAP and has been used to conduct a number of time-resolved studies (Mehrabi, Schulz, Agthe *et al.*, 2019 ▸; Mehrabi, Schulz, Dsouza *et al.*, 2019 ▸). In this work, we describe the application of an acoustic droplet addition to a fixed-target (‘drop-on-chip’ or DoC) approach (Kamps *et al.*, 2025 ▸) at Diamond Light Source (DLS) and Pohang Accelerator Laboratory (PAL) XFEL to modulate redox states in DtpB.

The differing timescales of X-ray exposure in SFX and SSX allow either ‘pristine’ triggered states or the dose response of triggered states in X-ray-sensitive enzymes to be probed, respectively. The ability to form a stable Compound I in microcrystals of DtpB at room temperature (Lučić, Svistunenko *et al.*, 2020 ▸) enables SSX to be used to investigate the effect of feeding X-ray-generated electrons to a pre-formed ferryl heme state. Previously, we have used SSX to obtain a room-temperature dose series starting with the Fe(III) heme state of an A-type DyP: the *S. lividans* dye-type peroxidase *Aa* (DtpAa; Ebrahim *et al.*, 2019 ▸). During continuous exposure to X-rays, dose-resolved structures from 33 to 314 kGy were obtained, showing a dose-dependent elongation and subsequent breakage of an iron–water coordinate bond (Ebrahim *et al.*, 2019 ▸). A recent study with DtpAa reinterpreted these electron-density changes as dose-dependent occupancy variation between two specific water positions (Gorel *et al.*, 2025 ▸). Either interpretation indicates positional change of an active-site water upon X-ray-induced redox-state change to the heme iron. Thus, serial approaches at both synchrotrons and XFELs can be used to drive redox-state change *in crystallo*.

In the present work, we have conducted two complementary serial crystallography experiments with DtpB using a fixed-target approach. The first uses the recently developed drop-on-chip (DoC) method that ejects solution droplets of a ligand or reactant to individual apertures containing microcrystals on the fixed target (Kamps *et al.*, 2025 ▸). To establish whether this method is suitable to generate Compound I in a heme enzyme, we ejected concentrated H_2_O_2_ onto microcrystals before exposure to XFEL pulses at PAL XFEL. In the second experiment, we pre-formed Compound I and then proceeded to capture the dose response of the ferryl state using dose-resolved SSX. The work highlights heterogeneity of the Fe(IV)—O bond length across the subunits of the DtpB hexamer, whether it be on the initial formation using the DoC approach or through absorbed dose. Taken together, these data showcase complementary approaches to initiate peroxidase reactivity and modulate the redox state of heme enzymes *in crystallo*.

## 2. Materials and methods

### 2.1. Overexpression, purification and microcrystallization of DtpB

Heterologous overexpression of DtpB in *Escherichia coli* BL21(DE3) cells and purification were carried out as described previously (Lučić, Svistunenko *et al.*, 2020 ▸). The stock concentration was determined by absorbance spectroscopy using a Varian Cary 60 UV–Vis spectrophotometer and an extinction coefficient (ɛ) at 280 nm of 18 575 *M*^−1^ cm^−1^. From the stock, DtpB solution aliquots were removed and mixed [1:1(*v*:*v*)] in a microfuge tube with the precipitant solution, consisting of 125 m*M* MgCl_2_, 125 m*M* HEPES pH 7.5, 18% PEG 4000, to give a final volume of 200 µl and a DtpB concentration of 6.2 mg ml^−1^. Microcrystals grew to sizes of 10–30 µm within 48 h.

To generate Compound I in DtpB microcrystals, a stock solution of 10 m*M* H_2_O_2_ was added to a 200 µl slurry of microcrystals to a final concentration of 600 µ*M*, resulting in a colour change from brown to green [*i.e.* an oxidation-state change from Fe(III) to Fe(IV)]. The microcrystal suspension was then loaded onto the silicon fixed-target chip and mounted onto the beam for measurement. The time to load, mount and align the chip with the beam was ∼10 min, with data collection taking 45 min. Therefore, the post-soaking time varies between 10 and 60 min, with this time increasing as the measurement progresses. Note that Compound I forms within seconds and is stable for at least 2 h (Lučić, Svistunenko *et al.*, 2020 ▸).

### 2.2. Loading of microcrystals onto fixed-target silicon chips

Microcrystals of ferric and Compound I DtpB were loaded under humidity-controlled conditions onto glow-discharged fixed-target silicon chips with 10, 12 or 14 µm apertures. A total of 200 µl microcrystal suspension was loaded followed by removal of excess precipitant by vacuum. For the SSX dose series experiments the chips were loaded with Compound I DtpB microcrystals, placed into a holder and sealed on both sides with a square of Mylar film (6 µm thick), followed by removal from the humidity chamber and mounting on the beamline. For the DoC experiments, chips loaded with ferric DtpB microcrystals were placed into a holder and sealed with a Mylar sheet on only one side, leaving the other side of the chip exposed for delivery of H_2_O_2_ by a piezoelectric injector.

### 2.3. Fixed-target serial experiments

**Figure 2:**
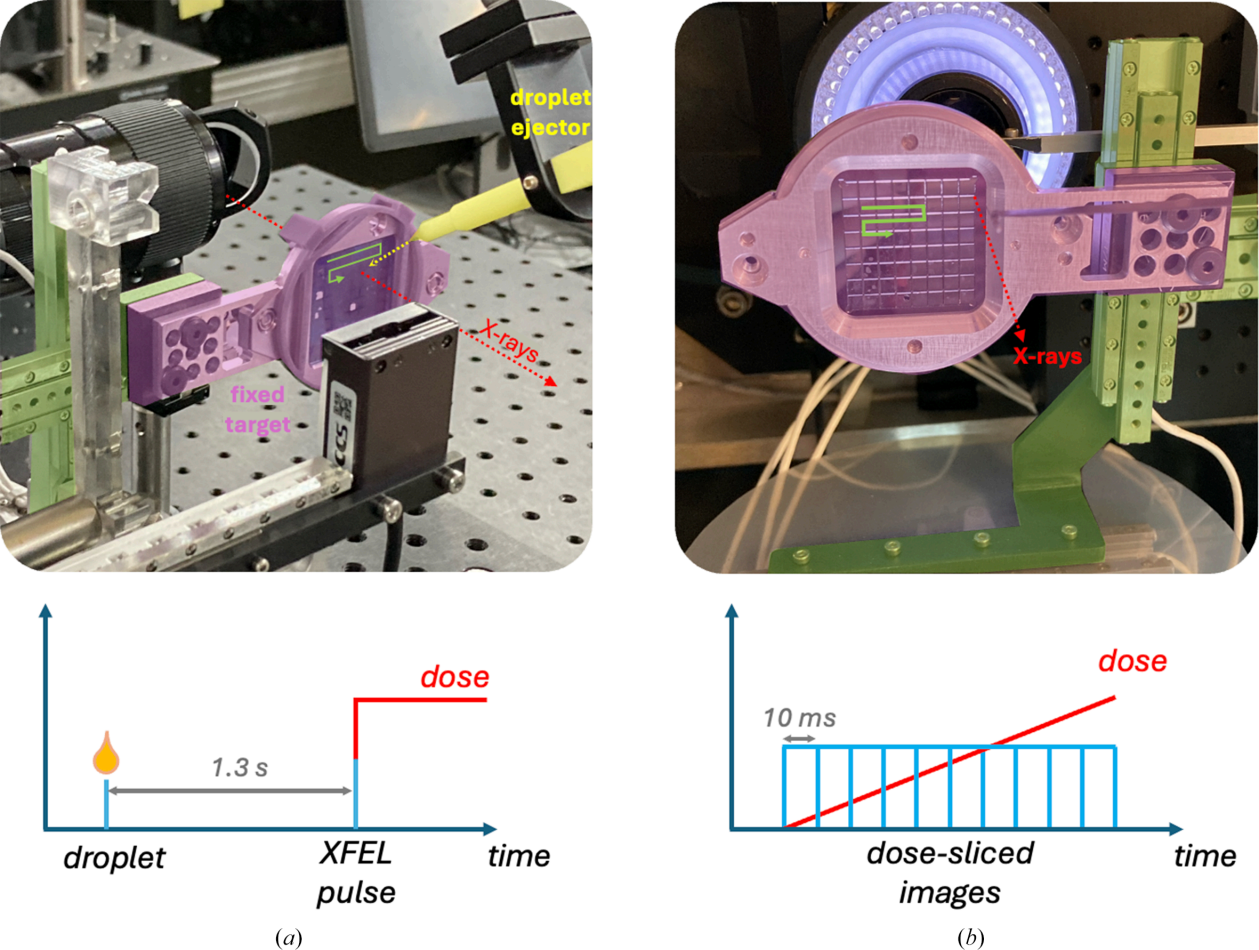
Experimental setup for serial fixed-target experiments using silicon chips at PAL and DLS. (*a*) Drop-on-chip at PAL utilizing the Autodrop pipette (shown in yellow). (*b*) Dose-series experiments at I24. The lower panel of each shows a timing schematic for each experiment including droplet delivery (PAL) and accumulation of dose (I24, DLS). Green arrows indicate the motion of the chip to bring apertures into position.

Identical motion hardware and control systems were used for SSX experiments at beamline I24 at Diamond Light Source (DLS) and SFX experiments at the Pohang Accelerator Laboratory (PAL) XFEL in South Korea. In brief, a three-axis stage (SmarAct) controlled by a Geobrick (Delta Tau) was used to position silicon fixed targets, or ‘chips’, in the X-ray beam (Sherrell *et al.*, 2015 ▸; Owen *et al.*, 2017 ▸). Creation of a virtual coordinate system allowed accurate and rapid positioning of the chips in the X-ray beam. Chips with 25 600 apertures were loaded with crystal slurry as described above and placed in a chip holder before being mounted on the three-axis stage using a kinematic mount (Jaho *et al.*, 2024 ▸). While a broadly similar approach was used at each source, differences in each setup and X-ray beam parameters for each experiment are detailed in Sections 2.4–2.6 and illustrated in Fig. 2 ▸.

### 2.4. Drop-on-chip experimental setup

Drop-on-chip (DoC) experiments were conducted at the PAL XFEL. The X-ray beam had an energy of 9.5 keV, a beam size of 2 × 2 µm, a 25 fs pulse duration and a repetition rate of 30 Hz. The DoC setup comprised of a piezoelectric injector (Autodrop pipette from Microdrop Technologies) coupled to the Smaract three-axis stage in a similar approach to that used by Mehrabi, Schulz, Agthe *et al.* (2019 ▸). The ejection of a ligand-containing droplet onto the chip is synchronized with the X-ray pulses through a TTL signal sent to the droplet controller 32.1 ms before each X-ray pulse. The piezoelectric injector is made of a glass capillary whose internal diameter defines the droplet size and volume: for the experiments described here a 30 µm inner-diameter capillary was used, resulting in single droplets of ∼100 pl of 100 m*M* H_2_O_2_ solution. Ejected droplets travel at about 1–2 m s^−1^ and the tip of the ejector is positioned 2 mm from the chip; thus, the time of flight of the droplets is <1 ms. After mounting the chip at the beamline, single droplets of H_2_O_2_ solution were added to each aperture on the chip at a defined time point before exposure to the XFEL pulse. The absorbed dose per exposure was estimated using *RADDOSE-XFEL* (Dickerson *et al.*, 2020 ▸) and is given in Supplementary Table S1. The time taken from droplet ejection to arrival in the fixed-target aperture is ∼1 ms and thus is negligible in comparison to the time delays used in this experiment. We note that the method used cannot rule out contamination, for example ejected droplets reaching the wrong aperture or overflowing the well. If there was such contamination, the mixing time point would be incorrect and that would mainly be true for short time points (tens to hundreds of milliseconds). For longer time points as presented here the error due to contamination becomes almost negligible. We have recently described in detail the DoC experiment with controls to avoid contamination (Kamps *et al.*, 2025 ▸).

### 2.5. SSX dose-series measurements

Experiments were conducted at beamline I24 at DLS using an X-ray energy of 12.8 keV, with a methodology essentially the same as that described in Ebrahim *et al.* (2019 ▸). Ten successive diffraction images with an exposure time of 10 ms were taken from each microcrystal within a fixed-target aperture at room temperature with no rotation, before moving to the next aperture. Diffraction images were then binned such that the first exposure from every position was merged to give the first dose point, the second image for the second dose point and so on for subsequent dose points. The absorbed diffraction-weighted dose for each image was estimated using *RADDOSE*-3*D* (Zeldin *et al.*, 2013 ▸) with dose increments corresponding to integer multiples of the total dose of 56 kGy accumulated within the exposure time of the first image.

### 2.6. Data processing and analysis

SSX dose-series data from DLS beamline I24 were processed using *DIALS* (version 1.14.12; Winter *et al.*, 2018 ▸) using the module *dials.stills_process* for indexing and integration and then *prime* for scaling and merging of observations, with the option for post-refinement enabled (Uervirojnangkoorn *et al.*, 2015 ▸). SFX data from PAL were processed using *xia*2.*ssx* (Beilsten-Edmands *et al.*, 2024 ▸). The starting model for refinement of all structures was the SFX structure of ferric DtpB (PDB entry 6yrj). Refinement was carried out using *REFMAC*5 (Murshudov *et al.*, 1997 ▸) within the *CCP*4 suite (Agirre *et al.*, 2023 ▸) with manual rebuilding in *Coot* (Emsley *et al.*, 2010 ▸). Non-crystallographic symmetry (NCS) restraints were applied during the refinement process. Structures were validated using *JCSG Quality Control Check* v.3.2 (https://qc-check.usc.edu/QC/qc_check.pl) and the PDB validation server as well as tools within *Coot*. The O atom of the oxo group was identified via omit *F*_o_ − *F*_c_ electron-density maps. Oxygen occupancy was assessed following the method previously described in Smyth *et al.* (2025 ▸). Refinement was performed using *REFMAC*5, and the occupancy of the bound O atom was systematically varied in increments of 0.05. After each refinement cycle, the *B* factor of the O atom was compared with that of the Fe-heme centre. The occupancy value at which the *B* factor of the O atom most closely matched that of the Fe-heme was considered optimal and was retained in the final model. We note that due to its tight coordination within the heme group the Fe atom may intrinsically have a lower *B* factor than the O atom, which could lead to an underestimation of the occupancy value derived from this method. Data-processing and refinement statistics for the DoC and dose-series experiments are reported in Tables 1 ▸ and 2 ▸, respectively.

## 3. Results

### 3.1. Initiation of the peroxidase reaction of DtpB using DoC mixing

**Figure 3:**
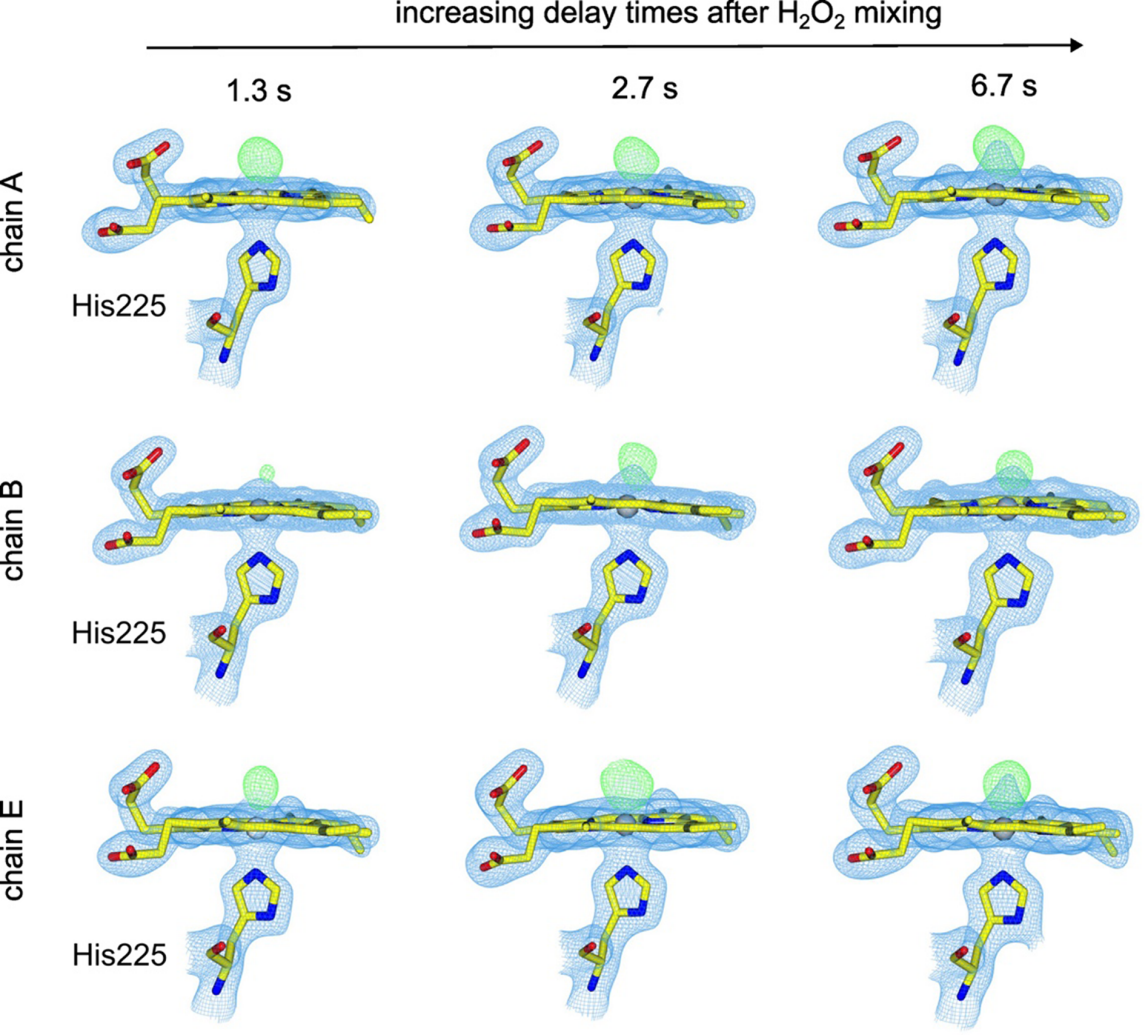
Time dependence of heme active-site changes in DtpB time-resolved DoC mixing experiments. Chains *A*, *B* and *E* of the DtpB hexamer were selected as representatives of three groups defined by their oxygen occupancy 1.3 s after H_2_O_2_ mixing: high-occupancy chains (*A* and *D*), low-occupancy chains (*B* and *C*) and intermediate-occupancy chains (*E* and *F*). Shown are 2*F*_o_ − *F*_c_ (blue, 1.5σ) and *F*_o_ − *F*_c_ electron-density maps (green, 3σ) at each time point after H_2_O_2_ mixing for the selected chains. In chain *A*, Compound I is nearly fully formed (O atom occupancy 0.8) before the first time point, followed by an increase to an occupancy of 1.0 by the final time point. In contrast, chain *B* exhibits the slowest rate of Compound I formation, with a small difference density peak observed at the first time point followed by a marked increase at the second time point (0.7). Chain *E* displays an intermediate formation rate between that of chains *A* and *B*, with an O atom modelled with a 0.65 occupancy at the first time point and 1.0 at the final time point.

**Figure 4:**
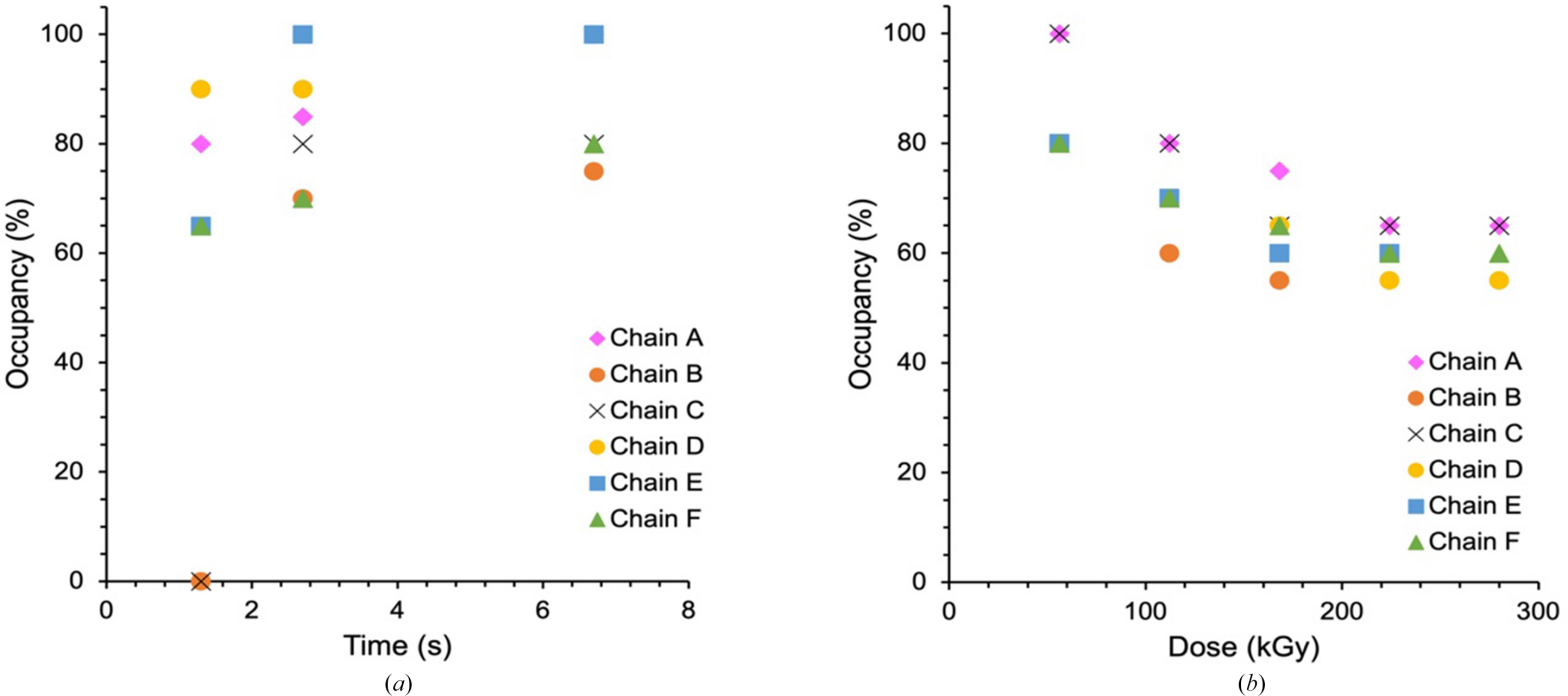
Occupancy of the iron-coordinated O atom versus post-mixing time and accumulated dose for each chain of the DtpB hexamer. (*a*) Time-dependent change in O-atom occupancy in individual DtpB chains over the time course of data collection shown in Fig. 3 ▸. (*b*) Dose-dependent changes in O-atom and H_2_O occupancy for the initially iron-coordinated O atom (Fig. 5 ▸).

Previous SFX measurements with fixed-target chips loaded with DtpB microcrystals soaked with H_2_O_2_ have shown that the active sites in all chains of the hexamer are able to interact with H_2_O_2_ to generate a Fe(IV)=O Compound I species. Notably, significant variability in the Fe–O distance following reaction with H_2_O_2_ across all heme sites in the hexamer was reported, with the shortest Fe—O bond length of 1.65 Å reported for chain *A* and the longest bond length of 1.89 Å for chains *B* and *C* (Lučić, Svistunenko *et al.*, 2020 ▸). Knowing that Compound I can be generated *in situ* by mixing microcrystal slurries with H_2_O_2_, and that the ‘resting state’ has an empty distal heme face (Fig. 1 ▸*b*), we tested whether we could follow Compound I formation in a time-resolved manner using DoC mixing. Microcrystals of DtpB in the resting ferric state were loaded within fixed targets under aerobic conditions as described and H_2_O_2_ was acoustically delivered to each aperture prior to XFEL pulses as described above at three time points, with all structures refined to 1.96 Å resolution (Table 1 ▸). Here, we note that we are not able to completely rule out aperture-to-aperture contamination by peroxide in these experiments and so the time points should not be considered strictly as reaction kinetics but instead as structural snapshots illustrating steps in the reaction. Further details regarding this potential issue are provided by Kamps *et al.* (2025 ▸). For these experiments, both the deadtime between droplet ejection and impact on the fixed target (∼1 ms) and diffusion through the crystal (tens to hundreds of milliseconds) are small in comparison to the eventual time points used. At the shortest time point explored (1.3 s), a *F*_o_ − *F*_c_ difference map peak appeared on the distal side of the heme Fe in all six heme sites, which would be consistent with the formation of an Fe(IV)—O species, *i.e.* Compound I (Fig. 3 ▸). Notably, the size of the electron-density feature (*i.e.* occupancy of the O atom) and its distance from the Fe atom were variable between the chemically identical heme groups in each chain of the hexamer. For example, at 1.3 s the occupancy of the O atom varied between 0 and 0.9, with an occupancy of 0.8 in chain *A*, while in chain *B* the electron-density feature was sufficiently weak that an O atom could not be reasonably modelled (Figs. 3 ▸ and 4 ▸*a*). These data suggest that Compound I had not yet fully formed in all of the protein molecules in the crystal after 1.3 s and that there are differences in the rate of its formation between the different heme sites within the hexamer, a feature that could be linked to the variation in Fe—O bond lengths previously identified. At the 2.7 s time point, the strength of the difference map feature and the occupancy of the modelled O atom increase such that the mean occupancy is 0.83 and the O atom can be modelled in every chain (Fig. 4 ▸*a*). At the longest time point of 6.7 s, we observed full-occupancy O atoms in chains *A*, *D* and *E*, with the lowest occupancy being 0.75 in chain *B* (Figs. 3 ▸ and 4 ▸*a*). When O atoms were added to the model and refined, the Fe—O distances were variable, in a similar manner to our previous SFX structure for the Compound I state achieved by soaking crystals in H_2_O_2_-containing solution over much longer time scales (Lučić, Svistunenko *et al.*, 2020 ▸).

### 3.2. Multiple serial structures generated through X-raydose-response of Compound I in DtpB

We next sought to investigate the effect of increasing X-ray dose on the preformed Fe(IV)=O Compound I state of DtpB at room temperature. Fe(IV)-containing enzymes are notoriously sensitive to electronic state changes upon X-ray exposure, largely because of the generation of solvated photoelectrons from radiolysis of water molecules within the hydrated protein crystal (Meharenna *et al.*, 2010 ▸). Our approach was very similar to that of our previous study examining the effect of dose on the ferric form of DtpAa (Ebrahim *et al.*, 2019 ▸). Structures were determined with a dose interval of 56 kGy, with the lowest dose-point structure being determined at a resolution of 1.75 Å (Table 2 ▸). As the dose series progressed, the resolution diminished as a consequence of global radiation damage, reaching 1.92 Å after 224 kGy and 2.19 Å after 336 kGy (Table 2 ▸). The loss in resolution for the final two dose points from this experiment (392 and 448 kGy) was such that they were not used for structural analysis as changes around the Fe—O group were no longer tractable. Data-processing and refinement statistics are reported in Table 2 ▸.

The lowest dose (56 kGy) structure revealed formation of Compound I, in agreement with our previously determined SFX structure where crystals in batch had been soaked with hydrogen peroxide in the same way prior to loading onto the fixed target (Lučić, Svistunenko *et al.*, 2020 ▸). Similarly to the previous study, we observed variability in the Fe—O bond length (1.65–1.89 Å) between the chains in the hexamer. As expected based on a previous study (Ebrahim *et al.*, 2019 ▸), the structure at this dose point likely represents a partially reduced heme state, due to the dose (56 kGy) already accumulated in determining the first structure using synchrotron radiation, and this is consistent with the observed differences between the damage-free SFX structure and the first dose point of the dose series.

**Figure 5:**
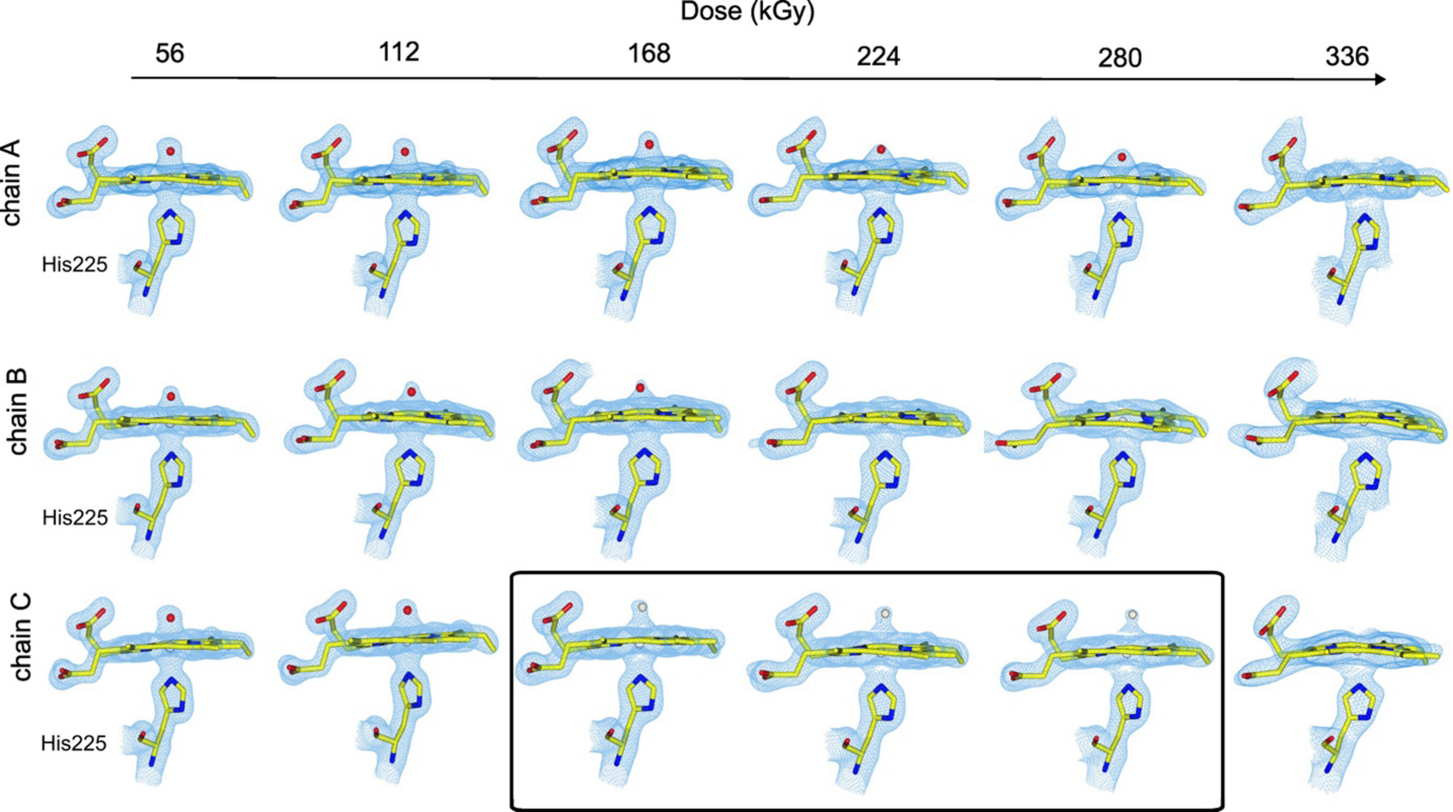
SSX dose-dependent structural changes of Compound I [Fe(IV)=O] in DtpB. 2*F*_o_ − *F*_c_ (blue mesh, 1.5σ) maps of the heme active site for selected chains are shown, where three distinct behaviours can be observed: (i) a gradual loss of electron density around the Fe—O bond accompanied by a slow increase in Fe–O distance (chains *A*, shown, and *D*, not shown), (ii) a rapid loss of electron density around the Fe—O group (chain *B*) and (iii) a gradual loss of electron density with a pronounced elongation of the Fe—O bond to ∼2.5 Å (chains *C*, shown, and *E* and *F*, not shown), consistent with conversion from a Compound I-like species to a water-bound state (chain *C*; boxed area). O atoms are shown as red spheres and water molecules as white spheres.

As the X-ray dose increases, the occupancy of the modelled O atom decreases consistent with reduction of Compound I to the five-coordinate ferric form of the enzyme and likely further reduction to the five-coordinate ferrous state (expected to be highly structurally similar to the ferric state). The mean O-atom occupancy in the heme pocket drops rapidly in all chains throughout the first three doses and then plateaus at 0.65–0.60 until the O atom can no longer be reliably modelled due to loss of resolution arising from global radiation damage (Fig. 4 ▸*b*). The dependency of the mean Fe—O distance upon X-ray dose does not follow a clear pattern and is not well resolved due to the dose-dependent loss of resolution (Supplementary Fig. S1). A more granular analysis unexpectedly revealed quite different behaviour within the chains of the hexamer (Fig. 5 ▸). In some chains the density corresponding to the O atom disappeared, while in others it stabilized at a particular fractional occupancy and elongated distance from the heme iron (Fig. 5 ▸, chain *C*). This could indicate the presence of a water molecule, generated from the breaking of the Fe—O bond as reduction occurs, and is less susceptible to further dose increments at this stage, remaining trapped within the heme pocket. Thus, in chain *C* a water was modelled for doses of 168, 224 and 280 kGy (Fig. 5 ▸). In chain *B* the O atom was modelled at the first three dose points and in chains *A* and *D* it remained for five dose points (280 kGy). Finally, we also considered whether an elongation of the Fe–O bond was present across the dose series. However, while substantial variation was observed in this bond, we note that bond-length changes were difficult to disentangle from dose-dependent loss of resolution and changes to occupancy, making obtaining tractable bond-distance information challenging.

## 4. Discussion

In this work, we have demonstrated two different methods of modulating the oxidation state of a heme peroxidase using serial crystallography. DoC mixing successfully generated the high-valence Fe(IV) state within microcrystals. Intriguingly, this process appeared to proceed at different rates in the different chains of the homohexameric protein. While the reaction clearly proceeds within the crystals on a timescale of seconds, we interpret specific time points with caution, as under the experimental conditions used we cannot completely exclude the possibility of some contamination of adjacent apertures in a chip during substrate addition to the targeted aperture. In our experimental design the volume of the added solution is much smaller than the capacity of the aperture, so we may reasonably expect contamination, if it exists, to be only minor. Here, we simply note that any such contamination of adjacent apertures would lead to a blurring of time resolution and precise time points should therefore be interpreted with caution. Future DoC experiments will aim to use a checkerboard method where alternate apertures do not have liquid added and are treated as a control, providing a ground-state control structure. For the reduction of Compound I by X-ray-generated electrons via the dose series of datasets the data are most consistent with an apparent two-electron process whereby Fe(IV)=O is reduced to the ferric resting state without populating the one-electron-reduced Compound II. This is wholly consistent with solution kinetics data, where Compound II is not detected (Lučić *et al.*, 2022 ▸).

A challenge in X-ray crystallography experiments is that at typical resolutions (1.5–2.5 Å) water molecules, OH^−^ species or O atoms appear almost identical in the electron-density maps, with differences between them only sometimes resolvable at the highest resolutions (better than 1.2 Å) or by using neutron crystallography (Kwon *et al.*, 2016 ▸). It is not possible therefore in data from the dose-series experiments to explicitly assign a point at which a dissociated O atom from the Fe(IV)-oxo group could be best described as a water molecule. However, our analysis based on Compound I of DtpB undergoing an apparent two-electron reduction to the Fe(III) ‘resting state’ rather than sequential single-electron reductions (Lučić *et al.*, 2022 ▸) suggests that in all cases we are observing a mixture of O atom and water in the distal heme pocket. Therefore, the apparent increase in Fe–O distance is likely due to a decrease of the O population (Compound I) and an increase of the water population, which will eventually be eliminated on returning to the fully Fe(III) ‘dry’ state (Lučić, Svistunenko *et al.*, 2020 ▸).

DtpB exists as a functional homohexamer in solution with no chemical differences in protein or cofactor composition (Lučić *et al.*, 2024 ▸). An intriguing observation from both of our experimental approaches was that the response of each of the heme centres in the hexameric protein was different. Such differences persisted whether the experiments were to form Compound I by rapid mixing of microcrystals with H_2_O_2_ or to investigate the effect of X-rays upon preformed Compound I. There has been no indication of different reactivities in solution kinetics data of this enzyme and so we investigated the possibility that the different environments of the heme centres or different protein dynamics within the crystalline lattice could cause the observed variation. We did not observe any substantive differences between the structures of each active site and the mean *B* factors across the chains vary between 35 and 38 Å^2^, suggesting that differences in the dynamics of the different chains are rather small and are unlikely to explain the substantial differences in their behaviour (Supplementary Table S2).

Analysis of solvent channels within the lattice revealed a somewhat different exposure to the bulk-solvent channels that run through the crystals. This allows us to hypothesize that the effectiveness of soaking of H_2_O_2_ as well as exposure to solvated photoelectrons generated from radiolysis of water molecules could be influenced by the accessibility to the bulk-solvent channels for any heme centre. Soaked reagents such as H_2_O_2_ can be assumed to enter the crystal lattice largely through the bulk-solvent channels that run throughout it, and subsequently to migrate from these large water-filled channels to any heme centre. The output bottleneck radius, the narrowest point of the main solvent channel that provides access to each heme in the DtpB hexamer, was computed to be 6.53 Å using the program *LifeSoaks* (Pletzer-Zelgert *et al.*, 2023 ▸). This radius is much larger than the projected radius of a H_2_O_2_ molecule (1.7–1.8 Å) and consequently would not be expected to hinder diffusion within the crystal to either heme pocket.

Similarly, reduction of metal centres in response to X-rays primarily occurs through the formation of solvated photoelectrons from the X-ray radiolysis of water, with these photoelectrons being formed in large numbers within the water-filled solvent channels (Shelley & Garman, 2024 ▸). We have previously pointed out that there is a hydrogen-bonding network from the Fe=O unit in Compound I of DtpB to the protein surface and hence to bulk solvent via Arg243 and the heme propionate 6 (Lučić, Svistunenko *et al.*, 2020 ▸), which would provide a feasible route for electron (and proton) transfer from solvent to the heme centre. A recent computational study has highlighted the possibilities of conserved pre-organized electrostatic funnels and solvent channels amongst peroxidase members that can draw protons towards the heme edges (Suardíaz *et al.*, 2025 ▸). Fluctuations in these channels could result in the heterogeneity that we observe at the heme sites between the different chains of the hexamer.

Future developments may make it more straightforward to analyse heterogeneity in reaction progress within the different monomers of the crystal structure. For example, UV–visible spectroscopy of this protein system would reveal the relative proportions of different redox states/intermediates within the crystal, although it is not possible to resolve signals from the different monomers and only the average spectrum is observed. Conducting such spectroscopic measurements from fixed targets is highly challenging, because of the nature of the crystal apertures and the crowded beamline environment, particularly when the droplet injection system is present, but nevertheless is under development by us. Molecular dynamics studies could in principle reveal differences in solvent accessibility of the different monomers or differences in dynamics and could be informative and implemented in future work.

More broadly, the experimental time points required to observe Compound I formation (several seconds) are very much longer than timescales for the same reactions in solution kinetics studies. Diffusion times for H_2_O_2_ from the crystal surface to the interior are estimated as being several tens of milliseconds for crystals of this size (Schmidt, 2013 ▸). Most of the increase in reaction times must therefore be due to other factors, for example changes to enzyme dynamics in the crystalline lattice, or perhaps some of the added peroxide reacts with other protein molecules on its way to the core of the crystal.

## 5. Conclusions

We have demonstrated that the oxidation state of a heme peroxidase in crystals can be effectively manipulated within fixed targets using serial crystallography. The DoC method successfully initiated enzyme reactivity within crystals by mixing microcrystals with H_2_O_2_ on the fixed target. This methodology is applicable to a wide range of time-resolved SFX and SSX studies on peroxide-dependent enzymes. We also demonstrate the utility of multiple serial structure dose-series experiments to modulate the redox states of peroxidase active sites beginning with the Fe(IV) state. Unexpectedly, the six chemically identical protein chains in the homohexamer of DtpB behave differently both in the rate and extent of the formation of Compound I and in their response to X-ray irradiation. This demonstrates the importance of the crystalline lattice and understanding the role of solvent/electrostatic channels for time-resolved or dose-resolved crystallographic studies at room temperature.

## 6. Related literature

The following reference is cited in the supporting information for this article: Gurusaran *et al.* (2014 ▸).

## Supplementary Material

PDB reference: DtpB Compound I, 56 kGy dose, 8pws

PDB reference: 112 kGy dose, 8pwy

PDB reference: 168 kGy dose, 9ewi

PDB reference: 224 kGy dose, 9ewj

PDB reference: 280 kGy dose, 9fb7

PDB reference: 336 kGy dose, 9fb9

PDB reference: 392 kGy dose, 9fba

PDB reference: 448 kGy dose, 9fbc

PDB reference: DtpB mixed with H_2_O_2_, 1.3 s delay, 9fbz

PDB reference: 2.7 s delay, 9fc0

PDB reference: 6.7 s delay, 9fc1

Supplementary Tables and Figures. DOI: 10.1107/S2059798326001658/xh5061sup1.pdf

## Figures and Tables

**Table 1:** Data-collection, analysis and refinement parameters for the DoC mixing experiment of DtpB with H_2_O_2_ conducted at the PAL XFEL Values in parentheses are for the outer shell.

Time delay (s)	1.3	2.7	6.7
Chips used	3	3	3
Wavelength (Å)	1.305	1.305	1.305
Effective absorbed X-ray dose (kGy)	0	0	0
No. of crystals	23641	25466	19553
*a*, *b*, *c* (Å)	86.9, 121.7, 199.4	86.9, 121.9, 200.2	86.9, 121,7, 199.5
Resolution (Å)	35.28–1.96	52.99–1.96	40.09–1.96
No. of reflections	144460	145256	144425
CC_1/2_	0.99 (0.23)	1.0 (0.23)	1.0 (0.25)
Multiplicity	165.8 (144.3)	152.7 (124.3)	116.3 (95.9)
Completeness (%)	100 (100)	100 (100)	100 (100)
Wilson *B* factor (Å^2^)	31.3	30.5	26.8
*R*_work_	0.167	0.178	0.200
*R*_free_	0.198	0.207	0.230
R.m.s.d., bond lengths (Å)	0.013	0.013	0.015
R.m.s.d., bond angles (°)	1.73	1.73	2.06
ESU based on *R*_free_ (Å)	0.127	0.132	0.129
ESU based on ML (Å)	0.116	0.121	0.119
Solvent atoms	710	687	680
Ramachandran most favoured (%)	98	98	98
PDB code	9fbz	9fc0	9fc1

**Table 2:** Data-collection, processing and refinement statistics for the X-ray dose-series data measured at beamline I24 for Compound I DtpB Note that the overall multiplicity value increases as a function of the absorbed dose due to the loss of higher resolution reflections from the dataset.

Chips used	2	2	2	2	2	2	2	2
Wavelength (Å)	0.9686	0.9686	0.9686	0.9686	0.9686	0.9686	0.9668	0.9686
Effective absorbed X-ray dose (DWD, kGy)	56	112	168	224	280	336	392	448
No. of crystals	23518	22905	22404	21858	21216	19785	18590	16846
*a*, *b*, *c* (Å)	86.7, 121.8, 199.5	86.8, 121.8, 199.5	86.8, 121.9, 199.5	86.8, 121.9, 199.6	86.9, 121.9, 199.6	86.7, 121.6, 199.0	87.0, 122.0, 199.6	87.0, 122.0, 199.6
Resolution (Å)	40.05–1.75	40.04–1.80	40.9–1.83	40.07–1.92	40.09–2.02	40.14–2.19	40.12–2.40	40.13–2.72
No. of reflections	212573	195649	186481	161825	139296	109752	83736	57840
*R*_split_	0.142 (0.794)	0.134 (0.743)	0.130 (0.736)	0.124 (0.705)	0.126 (0.567)	0.132 (0.748)	0.126 (0.927)	0.115 (0.782)
CC_1/2_	0.98 (0.50)	0.98 (0.53)	0.98 (0.55)	0.98 (0.57)	0.99 (0.57)	0.99 (0.53)	0.99 (0.46)	0.99 (0.52)
Multiplicity	64.3 (9.8)	68.1 (10.3)	70.7 (11.0)	78.3 (12.5)	85.2 (9.9)	95.4 (10.5)	113.3 (10.6)	140.5 (12.3)
Completeness (%)	100 (100)	100 (100)	100 (100)	100 (100)	100 (100)	100 (100)	100 (100)	100 (100)
Wilson *B* factor (Å^2^)	16.5	17.6	17.8	18.8	19.40	21.84	28.05	34.57
*R*_work_	0.197	0.195	0.200	0.206	0.228	0.246	0.242	0.237
*R*_free_	0.220	0.219	0.230	0.239	0.253	0.267	0.267	0.281
R.m.s.d., bond lengths (Å)	0.014	0.014	0.015	0.019	0.014	0.014	0.013	0.017
R.m.s.d., bond angles (°)	1.921	1.927	2.069	2.458	2.055	2.174	2.035	2.588
ESU based on *R*_free_ (Å)	0.106	0.114	0.126	0.146	0.173	0.224	0.281	0.401
ESU based on ML (Å)	0.165	0.170	0.190	0.232	0.288	0.385	0.454	0.620
Solvent atoms	457	395	339	240	69	10	3	2
Ramachandran most favoured (%)	98	98	98	97	97	96	96	94
PDB code	8pws	8pwy	9ewi	9ewj	9fb7	9fb9	9fba	9fbc

## Data Availability

Crystallographic coordinates and structure factors have been deposited in the Protein Data Bank.
