# Supplementary material for: Generation and modulation of catalytically relevant states of a dye-decolourizing peroxidase using time-resolved serial femtosecond crystallography with drop-on-chip mixing and X-ray-driven reduction
Source: Acta Crystallogr D Struct Biol. 2026 Apr 7;82(Pt 5):434–45. doi: 10.1107/S2059798326001658 (PMC13133994; doi:10.1107/S2059798326001658)
Supplement: Supplementary file 1 [file d-82-00434-sup1.pdf]

STRUCTURAL
BIOLOGY

Volume 82 (2026)

Supporting information for article:

Generation and modulation of catalytically relevant states of a dye-decolourizing peroxidase using time-resolved serial femtosecond crystallography with drop-on-chip mixing and X-ray-driven reduction

Marina Lučić, Johan Glerup, Pierre Aller, Danny Axford, Nicholas Devenish, Jaehyun Park, Anastasiia Shilova, Arturo Landeros de la Isla, Richard W. Strange, Tiankun Zhou, Robin L. Owen, Jonathan A. R. Worrall and Michael A. Hough

Table S1 Dose calculations for DtpB at PAL and DLS. Diffraction Weighted Dose (DWD) was calculated using RADDOSE-3D (Zeldin *et al.*, 2013)

PAL RADDOSE-3D calculations assume 1x10^{11} photons per unattenuated pulse. RADDOSE-3D style average dose whole crystal (RD3D) and average absorbed doses in the exposed region (ADER) were calculated using RADDOSE-XFEL.(Dickerson *et al.*, 2020)

Experiment	PAL	I24
Pulse duration/exposure time	25 fs	10 ms
X-ray energy	9.5 keV	12.8 keV
Pulse energy (pre-attenuation)	1 mJ	-
DWD	656 kGy	56 kGy
RD3D	785 kGy	-
ADER	674 kGy	-

Table S2 Average B-factors ($Å^2$) of individual DtpB chains from dose series and DoC structures.

Values were calculated using the custom PyMOL script average_b.py, which computes mean atomic B-factors for the specified selection.

	**Chain A**	**Chain B**	**Chain C**	**Chain D**	**Chain E**	**Chain F**
56 kGy	27.3	31.5	29.9	28.7	30.9	29.9
112 kGy	28.7	32.4	30.9	29.7	31.6	31.1
168 kGy	29.9	34.0	32.3	30.7	32.7	32.2
224 kGy	31.7	36.6	34.1	32.4	34.8	34.4
280 kGy	33.2	38.8	35.4	33.9	36.6	35.8
The336 kGy	36.0	41.3	36.9	35.4	37.9	37.7
392 kGy	33.8	40.9	35.4	32.0	34.7	34.9
448 kGy	53.9	63.6	58.5	55.4	58.8	56.7
1.3 s	36.6	42.0	39.9	38.4	38.7	38.8
2.7 s	37.5	42.0	40.3	38.0	39.2	39.6
6.7 s	34.8	39.5	37.7	36.0	36.5	36.9

Table S3 Occupancies and B-factors of Fe-coordinated O/H_2O in individual chains from dose series.

Values were obtained using Coot post Refmac5 refinement.

Dose	Chain A		Chain B		Chain C		Chain D		Chain E		Chain F	
	Occupancy	B factor	Occupancy	B factor	Occupancy	B factor	Occupancy	B factor	Occupancy	B factor	Occupancy	B factor
56 kGy	100	21.2	80	21.4	100	23.7	80	17.2	80	23.2	80	19.8
112 kGy	80	18.4	60	19.9	80	25	70	16.8	70	24	70	23.6
168 kGy	75	19.5	55	23.3	65	25.8	65	20.3	60	20.3	65	25.4
224 kGy	65	21.3	0	N/A	65	28.2	55	22.4	60	27.3	60	26
280 kGy	65	23.08	0	N/A	65	25.7	55	25.3	0	N/A	60	28.7
336 kGy	0	N/A	0	N/A	0	N/A	0	N/A	0	N/A	0	N/A
392 kGy	0	N/A	0	N/A	0	N/A	0	N/A	0	N/A	0	N/A
448 kGy	0	N/A	0	N/A	0	N/A	0	N/A	0	N/A	0	N/A

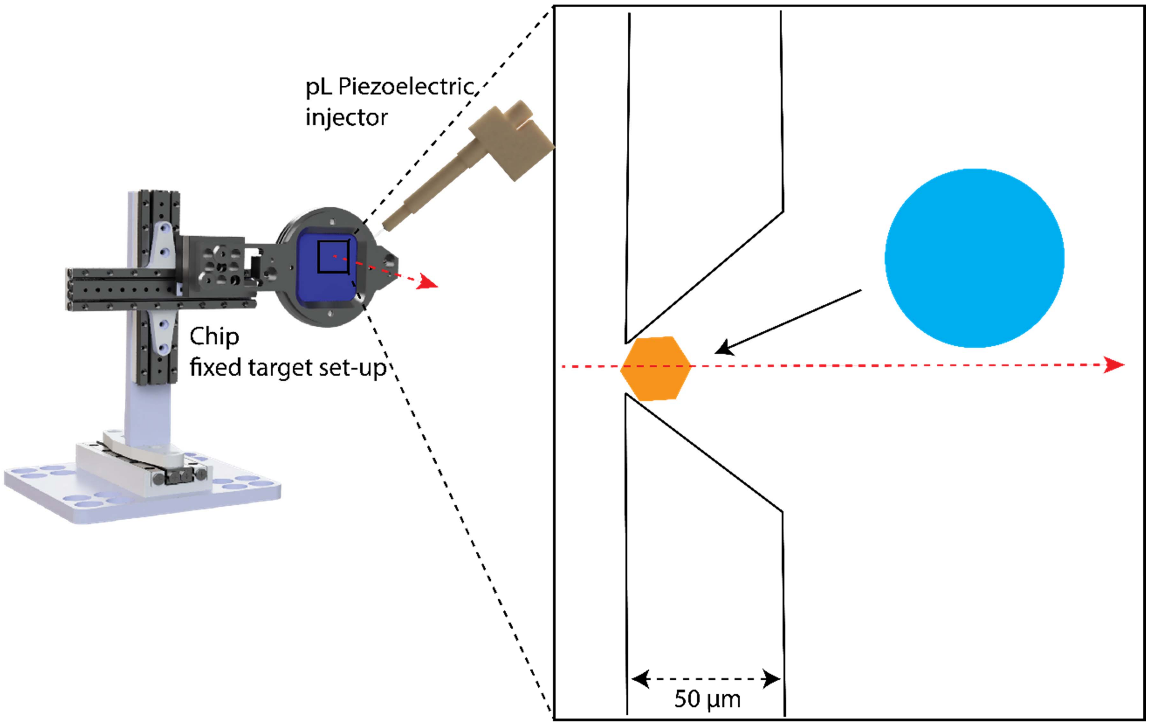

Figure S1 Schematic to scale showing a cross section of a chip well. A 20 µm crystal (yellow hexagon) is sitting in a well with a 15 µm aperture. The volume of a well is about 170 pL. The ejected droplet volume (blue circle) is 100 pL (~ 58 µm diameter). The X-ray beam is shown with a red dashed arrow.

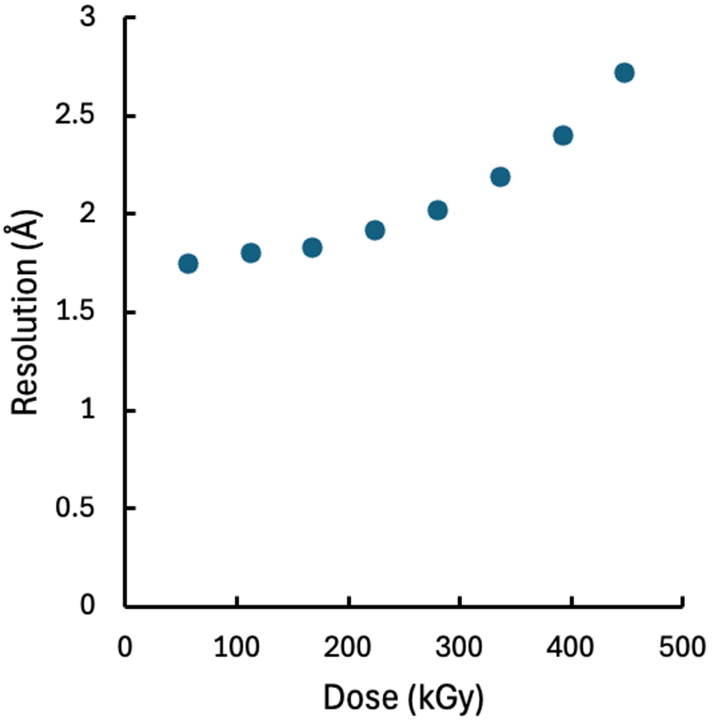

Figure S2 Resolution loss plotted as a function of accumulated dose during the dose series experiment. A progressive decrease in data resolution with increasing radiation exposure is observed.

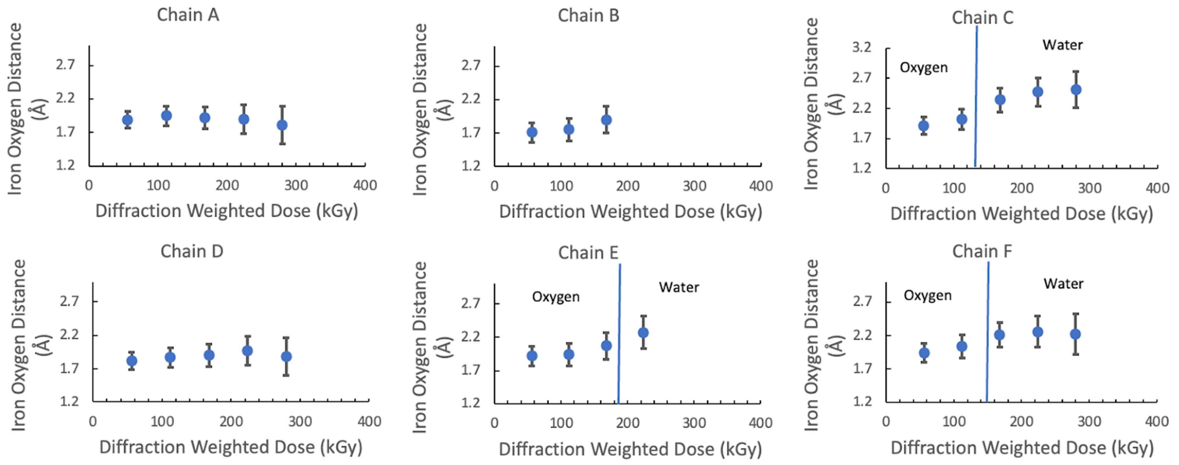

Figure S3 Distance between Fe and Fe-coordinated O atom/H_2O versus accumulated dose for each chain of the DtpB hexamer. Error bars represent average bond length errors calculated based on the two average coordinate errors in the direction of the bond determined as described in Gurasaran et al.

Supplementary references

Dickerson, J. L., McCubbin, P. T. N. & Garman, E. F. (2020). *Journal of Applied Crystallography* **53**, 549-560.

Gurusaran, M., Shankar, M., Nagarajan, R., Helliwell, J.R. & Sekar, K (2014). *IUCrJ* **1**, 74-81.

Zeldin, O. B., Gerstel, M. & Garman, E. F. (2013). *Journal of Applied Crystallography* **46**, 1225-1230.
